# Differential Behavioral and Neurobiological Effects of Chronic Corticosterone Treatment in Adolescent and Adult Rats

**DOI:** 10.3389/fnmol.2017.00025

**Published:** 2017-02-02

**Authors:** Jitao Li, Xiaomeng Xie, Youhong Li, Xiao Liu, Xuemei Liao, Yun-Ai Su, Tianmei Si

**Affiliations:** ^1^National Clinical Research Center for Mental Disorders (Peking University Sixth Hospital/Institute of Mental Health) and the Key Laboratory of Mental Health, Ministry of Health (Peking University)Beijing, China; ^2^Department of Psychiatry and Mental Health, North China University of Science and TechnologyTangshan, China

**Keywords:** adolescence, BDNF, corticosterone, depression, hippocampus

## Abstract

Adolescence is a critical period with ongoing maturational processes in stress-sensitive systems. While adolescent individuals show heightened stress-induced hormonal responses compared to adults, it is unclear whether and how the behavioral and neurobiological consequences of chronic stress would differ between the two age groups. Here we address this issue by examining the effects of chronic exposure to the stress hormone, corticosterone (CORT), in both adolescent and adult animals. Male Sprague-Dawley (SD) rats were injected intraperitoneally with CORT (40 mg/kg) or vehicle for 21 days during adolescence (post-natal day (PND) 29–49) or adulthood (PND 71–91) and then subjected to behavioral testing or sacrifice for western blot analyses. Despite of similar physical and neuroendocrine effects in both age groups, chronic CORT treatment produced a series of behavioral and neurobiological effects with striking age differences. While CORT-treated adult animals exhibited decreased sucrose preference, increased anxiety levels and cognitive impairment, CORT-treated adolescent animals demonstrated increased sucrose preference, decreased anxiety levels, and increased sensorimotor gating functions. These differential behavioral alterations were accompanied by opposite changes in the two age groups in the expression levels of brain-derived neurotrophic factor (BDNF), the phosphorylation of the obligatory subunit of the NMDA receptor, GluN1, and PSD-95 in rat hippocampus. These results suggest that prolonged glucocorticoid exposure during adolescence produces different behavioral and neurobiological effects from those in adulthood, which may be due to the complex interaction between glucocorticoids and the ongoing neurodevelopmental processes during this period.

## Introduction

Stressful life events constitute one of the environmental risk factors of several psychiatric disorders, such as depression, anxiety and schizophrenia (Hammen, 2005; Varese et al., 2012). Exposing laboratory animals to various stressors has been consistently found to produce behavioral and neurobiological alterations that resemble clinical findings with psychiatric patients (Willner, 2005; Sterner and Kalynchuk, 2010; McCormick and Green, 2013). However, stress does not necessarily produce adverse effects; under certain circumstances stress can promote performance and enable individuals to more effectively cope with the environment (McEwen, 2007; Lyons et al., 2010; Romeo, 2015; Sapolsky, 2015). Indeed, the consequences of stress in everyday life can be influenced qualitatively and quantitatively by multiple factors, including stressor types, duration, age, etc. (Joëls and Baram, 2009). One developmental stage that is of particular interest in this study regards adolescence, a critical window witnessing the typical onset of many psychiatric disorders (O’Donnell, 2011) and the continued maturation of stress systems (Romeo, 2013). It has been well-established that compared to adults, adolescent animals exhibited different hypothalamo-pituitary-adrenal (HPA) axis functions in that they showed prolonged exposure to stress hormones, including adrenocorticotropic hormone (ACTH) and corticosterone (CORT), in response to both acute and chronic stress (Romeo, 2013). This may be associated with the ongoing structural and functional remodeling of brain regions involved in stress responses, such as prefrontal cortex, hippocampus and amygdala (Spear, 2000). The behavioral and neurobiological consequences following the increased hormonal stress responses, however, still await systematic evaluation.

To investigate whether there exist adolescence-specific stress-induced alterations, studies should include adulthood as control and examine the effects of the same types of stressors in both adolescent and adult groups. There is a paucity of such studies so far (Green and McCormick, 2013; Romeo, 2013) and even these studies have produced contradictory behavioral results. Some studies found that adolescents are resilient to the stress-induced deleterious effects in adults. For instance, following the resident-intruder social stress, the defensive burying behavior was increased in adolescent rats, but decreased in adult rats (Bingham et al., 2011). Moreover, the same types of stressors resulted in depression-like behaviors (Toth et al., 2008) and cognitive dysfunction (Ricon et al., 2012; Zhang et al., 2016) in adult, but not adolescent, animals. In contrast, some studies found that adolescence is associated with increased stress vulnerability compared to adulthood, because the same types of stressors induced deficits in adolescent, but not adult animals (Morrissey et al., 2011). There were also studies reporting no age-related differences in terms of social and non-social anxiety, although adolescents exhibited an attenuated body weight increase and less habituation of CORT levels (Doremus-Fitzwater et al., 2009).

Repeated exposure to the major stress hormone, CORT, provides another means to induce depression-associated behavioral and neurobiological changes in animal models (Sterner and Kalynchuk, 2010). Compared to animal models with various physical and psychological stressors that affect individuals by activating the HPA axis to increase the CORT levels, this model explicitly manipulates the CORT concentrations in a quantitative manner, thus allowing to examine the role of CORT in stress-related alterations. Few studies have adopted this approach to directly compare adolescent and adult animals. One exception is a study showing that 1-week exposure to CORT (200 μg/ml in the drinking water) caused deficits in fear extinction in adolescent, but not adult, rats, indicating stress vulnerability in adolescents (Den et al., 2014). Therefore, more studies are needed to investigate whether CORT treatment could induce age-dependent changes in a variety of behaviors related with stress-related disorders, and if yes, what neural underpinnings are.

The hippocampus has been long implicated in the pathogenesis of stress-related psychiatric disorders. In this region, the stress hormone, CORT, binds to mineralocorticoid and glucocorticoid receptors and interacts with multiple cellular and molecular systems involved in synaptic plasticity, thereby influencing emotion and cognitive processing (McEwen, 2007). Both playing important roles in neurogenesis, dendritic remodeling and synaptogenesis, the brain-derived neurotrophic factor (BDNF) and glutamate neurotransmission have been extensively studied for their involvement in stress- and CORT-related behavioral alterations and structural remodeling. Clinical and animal studies have reported decreased BDNF levels in human patients or depressive animals and the normalization of its levels by antidepressant treatment, making BDNF a potential biomarker for mood disorders (Duman and Monteggia, 2006; Hashimoto, 2010). As for glutamate neurotransmission, various lines of evidence demonstrate that chronic stress leads to elevated glutamate activity and impaired synaptic plasticity, which may be associated with stress-induced cognitive deficits (Popoli et al., 2012) and that drugs targeting at the glutamatergic neurotransmitter system, such as ketamine, have great therapeutic potentials for mood disorders (Zanos et al., 2016). There also exist complex interactions between BDNF and glutamate transmission (Vásquez et al., 2014).

The aim of the present study was to examine the behavioral and neurobiological effects of chronic CORT administration in both adolescent and adult animals. A wide range of stress-related behaviors was assessed, including locomotor activity, exploration in the open field, sucrose preference, prepulse inhibition (PPI) and spatial learning in the Morris water maze. We also investigated the potential changes in expression levels of BDNF and ionotropic glutamate receptors in rat hippocampus following chronic CORT treatment in both age groups. Finally, recent evidence indicates the crucial roles of cell adhesion molecules, including nectin-3 (Wang et al., 2013) and neuroligin-2 (Kohl et al., 2015), in stress-induced behavioral alterations. Their expression levels were also examined in both age groups to investigate their potential involvement in CORT-induced effects.

## Materials and Methods

### Animals

Adolescent (*n* = 96, age 28–29 days, weighing 90 ± 5 g) and adult (*n* = 96, age 70–71 days, weighing 350 ± 20 g) male Sprague-Dawley (SD) rats were used in the present study. Animals were purchased from the Laboratory of Animal Science (Peking University Health Science Center, Beijing, China) and were given 1 week for acclimatization before any experimental procedures. They were housed 3–4 per cage in a controlled environment (23 ± 1°C; 35–55% relative humidity; fixed 12/12 h light/dark cycle, lights on at 08:00 h) with food and water *ad libitum*. All procedures were performed in accordance with the National Institute of Health’s Guide for the Use and Care of Laboratory Animals and were approved by the Peking University Committee on Animal Care and Use.

### Drugs and Pharmacological Procedures

CORT (Sigma, C2505) was dissolved in propylene glycol and stored at 4°C. Adolescent and adult rats were randomly divided into vehicle and CORT groups and received an intraperitoneal injection of either propylene glycol or CORT (40 mg/kg in vehicle) between 09:00 am and 11:00 am once daily for 21 days. The treatment dose and duration was chosen based on previous studies with adult animals (Sterner and Kalynchuk, 2010). The volume of injection was 2 ml/kg and 1 ml/kg for adolescent and adult animals, respectively. Body weights were recorded every other day during the treatment period.

### General Experimental Design

**Figure 1:**
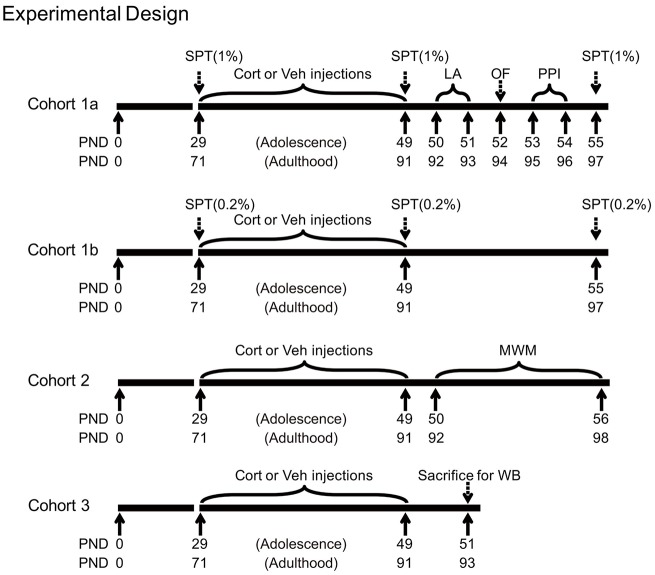
**Experimental timelines of chronic corticosterone (CORT) administration, behavioral and western blot procedures in three animal cohorts.** PND, postnatal day; Cort, corticosterone; Veh, vehicle; SPT, sucrose preference test; LA, locomotor activity; OF, open field; PPI, prepulse inhibition; MWM, Morris water maze; WB, western blot.

Shortly after the last injection, animals were subjected to various experimental procedures. Three animal cohorts were used, with the first two cohorts tested for behavioral effects and the third for stress-related neurobiological changes. The first cohort (*n* = 10 per group) was tested for sucrose preference (1% sucrose solution), locomotor activity, free exploration in the open field and PPI. We further tested an independent cohort of animals (*n* = 10 per group) for sucrose preference using the 0.2% sucrose solution to avoid the potential influence between the two concentrations of sucrose solutions. The 0.2% sucrose solution was chosen, because adolescents tend to show reduced preference to stimuli with low incentive values (Graaf and Zandstra, 1999; Spear, 2000), thus providing room for testing the potential CORT-induced alteration in both decreasing and increasing directions. As the spatial learning task in the Morris water maze consisted of consecutive training sessions in several days, this task was performed in the second cohort (*n* = 20 per group). The third cohort (*n* = 8 per group) was deeply anesthetized with pentobarbital 48 h after the last injection. Animals’ brain and blood samples were then collected for western blot and CORT analyses. Adrenal glands were also removed, dissected from fat and weighed. The timing of various procedures in these cohorts is shown in Figure 1.

### Corticosterone Analysis

Blood samples were collected in 1.5 ml ethylenediaminetetraacetic acid-coated microcentrifuge tubes. All samples were kept on ice and later centrifuged at 3000 rpm for 15 min at 4°C. Plasma was transferred to clean 0.2 ml Eppendorf tubes and stored at −80°C until use. Plasma CORT concentrations were measured using a CORT EIA kit (Cayman Chemical, Ann Harbor, MI, USA) as described previously (Regev et al., 2012).

### Behavioral Procedures

*Locomotor activity*, expressed as the total distance traveled by rats during 60 min, was recorded individually for each animal in a black soundproof chamber (40 × 40 × 65 cm) equipped with 5-W lamps in four sidewalls and an overhead video recorder.

*Open field*. The testing apparatus was a 100 × 100 × 50 cm box made of gray polypropylene and illuminated at 60 lux during testing. During the test, animals were individually placed in the corner of the box and allowed to freely explore the environment for 10 min. The time spent in the central field and the latency of the first entry to the central field were recorded as anxiety-related indices.

*The sucrose preference test* is used to assess anhedonia, a major symptom of depression, by determining animals’ preference for sucrose solution over tap water. Five hours before the test (at 15:00 pm), animals were single housed and water deprived, with free access to food. The test started at the beginning of the dark phase (at 20:00 pm) of the animals’ cycle, during which animals were presented with two bottles for 12 h, one with sucrose solution and the other with tap water. Liquid consumption (water and sucrose solution) was estimated by weighing the bottles before and after exposure to the animals. Sucrose preference percentage (%) = (sucrose solution consumption)/(water consumption + sucrose solution consumption) × 100. For each concentration, the procedure was conducted three times, i.e., before the first drug treatment (baseline), 24 h and 1 week after the last CORT treatment, respectively.

*Prepulse inhibition (PPI) of the acoustic startle response*, expressed as the reduction of the motor response to a loud acoustic stimulus (120 dB) by a preceding weak sound (3–12 dB above the background noise), is an index of sensorimotor gating (Braff and Geyer, 1990). The task was performed in SR-LAB startle boxes (San Diego Instruments, San Diego, CA, USA) as described previously (Li et al., 2013). Animals were acclimatized to startle chambers for 15 min on the day before testing. On the testing day, the experiment began with an acclimatization period of 5 min, followed by six consecutive pulse stimuli (the 120 dB white noise) in order to scale down the initial startle response to a stable plateau. The test session then began, consisting of eight types of trials: (i) pulse alone: the 120 dB white noise; (ii–iv) prepulse plus pulse: a prepulse stimulus of 3, 6 or 12 dB above the background noise (68 dB) was presented 100 ms before the onset of the pulse; (v–vii) prepulse alone: 3, 6 or 12 dB above the background noise; and (viii) no stimulus. Each stimulus was 20 ms in duration. Each trial type was presented 10 times in a pseudorandom order with the inter-trial interval (ITI) varying from 8 s to 22 s. The total test lasted approximately 34 min. The startle magnitude was calculated as the average response to pulse-alone trials. PPI was the percent decrease of the startle in pulse-alone compared to startle in prepulse-plus-pulse trials: %PPI = [1 − (startle response to (prepulse-plus-pulse) trial/ (startle response to pulse-alone) trial)] × 100.

*Spatial learning test* was performed in the Morris water maze (Vorhees and Williams, 2006). The swimming pool (185 cm in diameter, 45 cm in height, made from dark plastic) was filled with tap water (thermostatically controlled at 23 ± 1°C), which was made opaque by adding black ink. A circular platform (9 cm in diameter) was submerged 1 cm below the water surface. The test consisted of seven consecutive days of training for animals to acquire the location of the hidden platform, followed by a probe test on the 8th day. The pool was divided into four equal imaginary quadrants. The platform was located at the center of one quadrant, with its location fixed throughout the training phase. On each training day, rats received four trials, each starting from different locations. In each trial, an animal was placed in the pool with its nose facing the wall. Animals finding the platform within 60 s were allowed to sit on it for 15 s and those failing to do so were guided by an experimenter to the platform and then allowed to sit on it for 15 s. The ITI was 20 s. In the probe test, rats were placed in the pool without the platform to swim for 60 s and their swimming time in each quadrant was collected and analyzed. After swimming, animals were dried with a towel and put in a clean cage to avoid interaction with other animals. Spatial learning performance in the training phase was expressed as the mean escape latency to the platform over four trials in a training day. For the probe test, the percentage time animals swam in the target, adjacent and opposite quadrants were used as an indication of spatial memory.

### Western Blot

Using western blot, we examined the following protein levels in rat hippocampus. First, both types of CORT receptors (MR and GR) were examined for the neuroendocrine effects of chronic CORT administration. The mature BDNF and the precursor of BDNF (proBDNF) were measured for the BDNF signaling system. Ionotropic glutamate receptor subunits (NMDA receptor subunits: GluN1, GluN2A, GluN2B and their phosphorylated forms together with PSD-95, a postsynaptic protein that stably co-localizes with NMDA receptors; AMPA receptor subunits: GluA1 and GluA2) were used as markers for glutamate neurotransmission. Finally, two stress-related cell adhesion molecules (nectin-3 and neuroligin-2) were determined for potential alterations following chronic CORT treatment.

Forty-eight hours after last drug treatment, rats were deeply anesthetized with pentobarbital and their brains rapidly removed and dissected to obtain hippocampus as previously described (Gearhart et al., 2006). Tissue from individual rats was immediately homogenized on ice in ice-cold lysis buffer (137 mM NaCl, 20 mM Tris–HCl (pH 8.0), 1% NP-40, 10% glycerol, 1 mM PMSF, 10 mg/ml aprotinin, 1 mg/ml leupeptin, 0.5 mM sodium vanadate), sonicated, and centrifuged. The supernatants were stored at −80°C until required.

Samples containing 30 μg of protein were resolved by 12.5% (for BDNF) or 10% (for other proteins) sodium dodecyl sulfate polyacrylamide gel electrophoresis (SDS-PAGE) gels, and transferred electrophoretically to a polyvinylidene difluoride (PVDF) membrane (Millipore, Bedford, MA, USA). The PVDF membranes with the pore size being 0.2 μm for BDNF and 0.45 μm for other proteins containing the proteins of interest were then blocked with 5% non-fat milk diluted in Tris-buffered saline-Tween (TBST) (150 mM NaCl, 10 mM Tris-HCl (pH 7.5) and 0.1% Tween) for 1 h at room temperature and incubated overnight at 4°C in primary antibodies diluted in TBST containing 5% non-fat milk (MR: 1:2000, ab64457, Abcam; GR: 1:25,000, 3626–1, Epitomics; BDNF: 1:1000, ab108319, Abcam;GluN1: 1:2000, ab109182, Abcam; p-GluN1: 1:2000, 3384S, Cell Signaling; PSD-95: 1:1000, sc32290, Santa Cruz; GluN2A: 1:1000, 4205, Cell Signaling; pGluN2A: 1:2000, ab16646, Abcam; GluN2B: 1:1000, 4207S, Cell Signaling; pGluN2B: 1:1000, 4208, Cell Signaling; GluA1: 1:5000, 182011, Synaptic Systems; GluA2: 1:2000, MAB397, Millipore; Nectin-3: 1:5000, ab63931, Abcam; Neuroligin-2: 1:10,000, 129203, Synaptic Systems; β-actin: 1:20,000, 3700S Cell Signaling; actin: 1:1000, sc1616, Santa Cruz). The next day, membranes were rinsed three times with TBST (8 min each time) and incubated for 2 h with horseradish peroxidase-conjugated goat anti-rabbit or anti-mouse secondary antibodies (1:2500–20,000, Santa Cruz Biotechnology, Dallas, TX, USA) diluted in TBST. Following another three TBST rinses, proteins of interest were visualized using an ECL system (Pierce, Rockford, IL, USA) and Kodak XBT-1 film. The immunoreactive signals of the target proteins were quantified by densitometry and the values were corrected based on their corresponding β-actin levels. All results were normalized by taking the value of the vehicle group as 100%.

Considering that the western blot of MR showed double bands, we examined the specificity of the MR antibody using the corresponding blocking peptide (1:1000, ab74464, Abcam) and found that both bands were significantly blocked (see Supplementary Figure S1, together their expression levels were reduced by 60.01%). So both of them were included in data analyses.

### Statistical Analyses

All data are expressed as the mean ± SEM. Raw data values were checked for outliers, with any data point more than twice the standard deviation away from the group mean deemed outliers and removed from analyses. Comparisons of behavioral and neurobiological data between CORT and vehicle treatment within each age group were performed using independent-samples *t*-tests. For data acquired over a period of time, including body weight over the treatment period, distance traveled in the locomotor activity test, PPI levels over three prepulse types and spatial learning performance across training days, repeated measures analysis of variance (ANOVA) was used with treatment as a between-subject factor, testing day or time as a within-subject factor. The percentage time animals spent in the target, adjacent and opposite quadrants in the probe test of the spatial learning task was compared with each other using the Wilcoxon signed rank test. The significance level for all statistical tests was *P* < 0.05.

## Results

### Physical and Neuroendocrine Effects of Chronic CORT Treatment in Adult and Adolescent Animals

**Figure 2:**
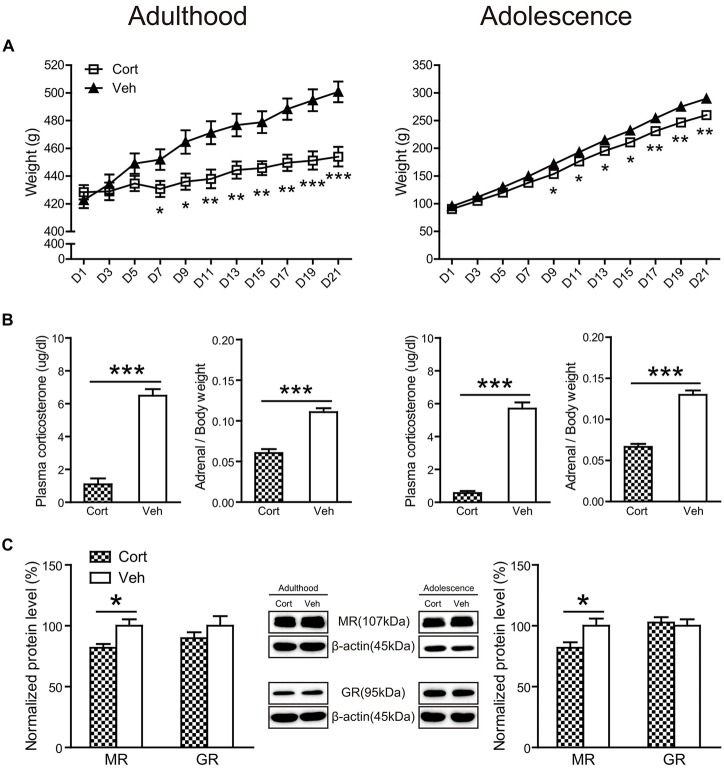
**Physical and neuroendocrine effects of Chronic CORT treatment in adult and adolescent animals. (A)** Body weight during the treatment period; **(B)** plasma CORT levels and adrenal weight; **(C)** hippocampal MR and GR expression levels. For the body weight data in both ages, *n* = 10 in the vehicle group and *n* = 8 in the CORT group. For the plasma CORT levels, sample sizes in the adult CORT, adult vehicle, adolescent CORT and adolescent vehicle groups were 8, 9, 7 and 8 respectively. For the adrenal weight, *n* = 10 per group in adult animals and in the adolescent vehicle group; *n* = 9 in the adolescent CORT group. For MR and GR levels, *n* = 8 per group for MR in adult animals, MR and GR in adolescent animals; for GR levels in the adult groups, *n* = 7 in the CORT group and *n* = 8 in the vehicle group. *p < 0.05, **p < 0.01, ***p < 0.001, compared with the vehicle group. Cort, corticosterone; Veh, vehicle.

Chronic CORT treatment in both adult and adolescent animals significantly decreased body weight throughout the treatment course (Figure 2A). For adult animals, repeated measures ANOVA of body weight revealed significant main effects of treatment (*F*_(1,16)_ = 7.86, *P* = 0.013) and day (*F*_(10,160)_ = 84.96, *P* < 0.001), as well as the treatment × day interaction (*F*_(10,160)_ = 19.51, *P* < 0.001). Examining the treatment effect on each day showed that animals receiving CORT treatment had significantly lower body weight than animals in the vehicle group from the seventh treatment day up to the end of the treatment (*P*s < 0.05). Similar results were found for adolescent animals. Significant main effects of treatment (*F*_(1,16)_ = 10.56, *P* = 0.005) and day (*F*_(10,160)_ = 1713.58, *P* < 0.001), as well as the treatment × day interaction (*F_(10,160)_* = 7.47, *P* < 0.001) were observed. Body weight in the CORT-treated adolescent animals started to show significant decrease from the ninth treatment day (*P*s < 0.05).

The relative weight of adrenal glands over body weight (Figure 2B) was significantly decreased by chronic CORT treatment in both adult (*t*_(17)_ = 7.43, *P* < 0.001) and adolescent (*t*_(18)_ = 9.93, *P* < 0.001) groups. Chronic CORT treatment also significantly reduced plasma CORT levels (Figure 2B) in both age groups (adulthood: *t*_(13)_ = 9.93, *P* < 0.001; adolescence: *t*_(15)_ = 12.35, *P* < 0.001).

The expression levels of mineralocorticoid (MR) and glucocorticoid receptors (GR) were examined in the hippocampus (Figure 2C). Exposure to chronic CORT treatment significantly reduced MR expression in the hippocampus of both adult and adolescent animals (adulthood: *t*_(14)_ = 2.90, *P* = 0.012; adolescence: *t*_(14)_ = 2.44, *P* = 0.028), whereas GR expression was not affected in either age group (*P*s > 0.31).

### Behavioral Effects of Chronic CORT Treatment in Adult and Adolescent Animals

#### Locomotor Activity

**Figure 3:**
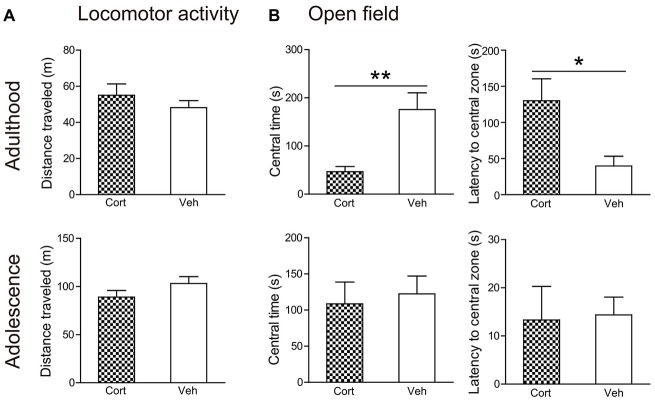
**Effects of chronic CORT treatment in adult and adolescent animals on locomotor activity and open field tests. (A)** Total distance traveled in the locomotor activity test. **(B)** Time spent in, and the latency to enter, the center of the open field. For the locomotor activity test, *n* = 8 in the CORT group and *n* = 10 in the vehicle group in both ages; for the open field test, *n* = 10 in the vehicle group in both ages, *n* = 9 and 8 in the CORT group in adult and adolescent animals, respectively. *p < 0.05, **p < 0.01, compared with the vehicle group. Cort, corticosterone; Veh, vehicle.

Locomotor activity (Figure 3A) was not altered by chronic CORT treatment in both adult and adolescent animals. The total distance traveled in 60 min was comparable in CORT- and vehicle-treated animals in both age groups (*P*s > 0.18). Analyses of distance traveled every 5 min using repeated measures ANOVA also failed to reveal significant main effects of treatment or the treatment × time interaction (*P*s > 0.35).

#### Open Field Test

Chronic CORT treatment induced differential effects in two age groups in the open field test (Figure 3B). CORT-treated adult animals were found to spend significantly less time in the central portion of the testing box (*t*_(17)_ = 3.39, *P* = 0.003) and to take significantly longer time to approach the central field (*t*_(17)_ = 2.81, *P* = 0.012). These effects were not evident in adolescent animals. The time spent in the central field and the latency to approach it was comparable in adolescent animals receiving CORT and vehicle treatment (*P*s > 0.73).

#### Sucrose Preference

**Figure 4:**
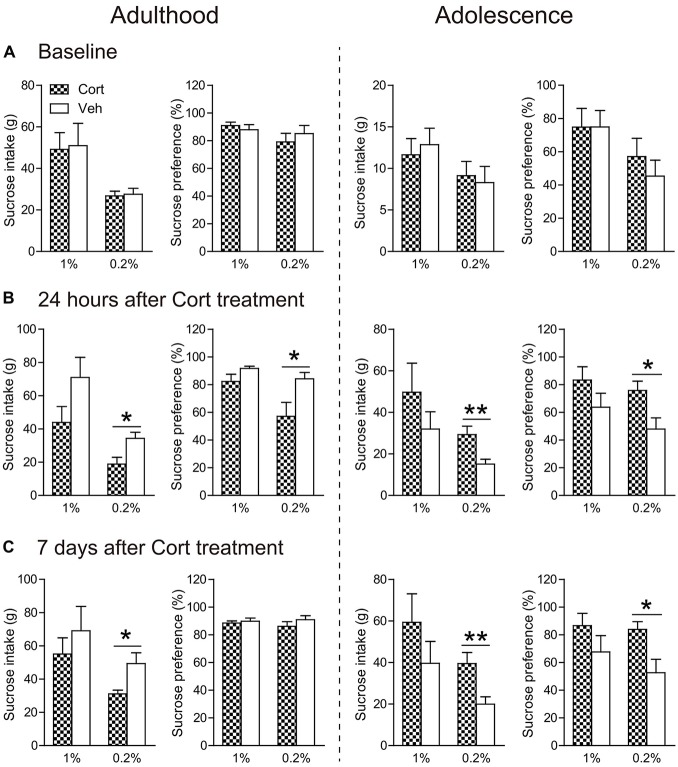
**Effects of chronic CORT treatment in adult and adolescent animals on sucrose intake and preference.** Measurements were recorded **(A)** before the first CORT treatment, **(B)** 24 h after and **(C)** 7 days after the last CORT treatment. Two animal cohorts were tested on 1% and 0.2% sucrose solution, respectively. For adult animals, *n* = 10 per group for 1% sucrose solution and *n* = 9 per group for 0.2% sucrose solution; for adolescent animals, *n* = 8 in the CORT group at both concentrations and *n* = 10 and 9 for 1% and 0.2% sucrose solution, respectively, in the vehicle group. *p < 0.05, **p < 0.01, compared with the vehicle group. Cort, corticosterone; Veh, vehicle.

Before treatment, all groups of animals had similar consumption of sucrose solution and sucrose preference, irrespective of the concentrations of the sucrose solutions (Figure 4A).

Twenty-four hours after chronic CORT treatment, age-dependent effects were observed (Figure 4B). When the concentration of sucrose solution was 1%, CORT-treated adult animals exhibited a tendency of decreased intake of sucrose solution compared to vehicle-treated animals (*t*_(17)_ = 1.75, *P* = 0.097), whereas adolescent animals receiving chronic CORT treatment seemed to show increased sucrose consumption, which however did not approach statistical significance.

With the 0.2% sucrose solution to which adolescent controls showed reduced preference than adults (Supplementary Figure S2c), the age-opposite effects were conspicuous in that sucrose consumption and preference were significantly decreased in CORT-treated adult animals (intake: *t*_(16)_ = 2.80, *P* = 0.013; preference: *t*_(16)_ = 2.45, *P* = 0.026), but increased in CORT-treated adolescent animals (intake: *t*_(15)_ = 3.08, *P* = 0.008; preference: *t*_(16)_ = 2.62, *P* = 0.019).

The age-opposite effects of chronic CORT treatment on 0.2% sucrose intake and preference even persisted after a 1-week withdrawal period (Figure 4C). Adult animals receiving CORT treatment still showed decreased sucrose intake compared to the vehicle animals (*t*_(16)_ = 2.67, *P* = 0.017), whereas adolescent animals receiving the same treatment again showed increased intake of and preference to the sucrose solution (intake: *t*_(15)_ = 3.12, *P* = 0.007; preference: *t*_(16)_ = 2.69, *P* = 0.017).

#### Prepulse Inhibition

**Figure 5:**
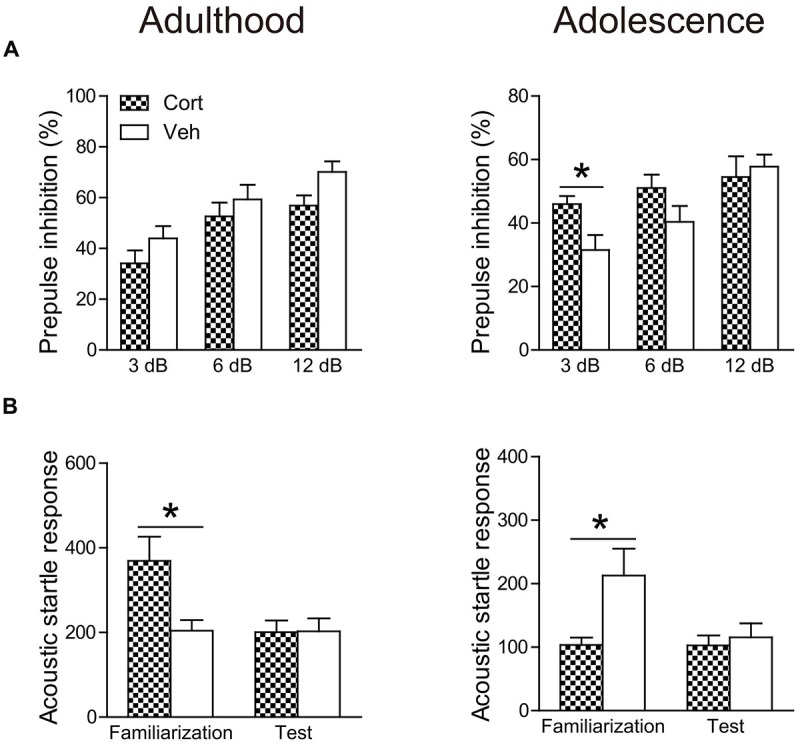
**Effects of chronic CORT treatment in adult and adolescent animals on PPI (A) and acoustic startle responses **(B)**.** Acoustic startle responses were recorded and averaged over six startle trials during the familiarization phase and over ten startle trials during the test session, respectively. *n* = 8 in the CORT group and *n* = 9 in the vehicle group for both ages. *p < 0.05, compared with the vehicle group. Cort, corticosterone; Veh, vehicle.

The age-dependent effects of chronic CORT treatment were also observed in PPI levels (Figure 5A). Repeated measures ANOVA in the adult animals did not reveal significant prepulse × treatment interaction (*F*_(2,30)_ = 1.52, *P* = 0.23). PPI levels were then collapsed across three prepulse levels to examine the treatment effect, which showed that chronic CORT treatment tended to decrease PPI levels, although this trend did not approach statistical significance (*t*_(15)_ = 1.49, *P* = 0.16). For adolescent animals, a significant prepulse × treatment interaction (*F*_(2,30)_ = 3.61, *P* = 0.039) was observed. The interaction was primarily driven by the significantly higher PPI levels in the CORT-treated animals relative to vehicle-treated animals when the prepulse intensity was low (*t*_(15)_ = 2.58, *P* = 0.021).

To examine whether the PPI changes were attributable to prepulse reactivity (Yee and Feldon, 2009), we compared the motor reactivity of animals in the vehicle and CORT groups during the prepulse-alone trials and the “no-stimulus” trials (Table 1). The “no-stimulus” trials were included as a baseline condition. The main effects of treatment (*P*s > 0.79) and the prepulse × treatment interaction (*P*s > 0.18) did not approach significance in adult or adolescent animals using repeated measures ANOVA. *A priori* between-group comparisons at each prepulse intensity did not show significant treatment effects, either (*P*s > 0.31).

**Table 1:** **The mean motor reactivity (in arbitrary units ± SEM) during the no-stimulus and prepulse-alone trials in the PPI test**.

	Adulthood		Adolescence	
Trial type	Corticosterone (*n* = 8)	Vehicle (*n* = 9)	Corticosterone (*n* = 8)	Vehicle (*n* = 9)
No stimulus	4.13 ± 0.81	4.67 ± 0.78	2.55 ± 0.37	2.46 ± 0.38
Prepulse 3 dB	3.65 ± 0.53	3.64 ± 0.33	2.34 ± 0.29	2.62 ± 0.31
Prepulse 6 dB	4.24 ± 0.61	3.89 ± 0.30	2.74 ± 0.45	2.67 ± 0.42
Prepulse 12 dB	4.51 ± 0.61	3.83 ± 0.52	3.12 ± 0.49	2.45 ± 0.40

The averaged startle response during the test session was not affected in either age group by chronic CORT treatment (Figure 5B). However, the averaged startle response during the familiarization phase in the two age groups was differentially regulated by chronic CORT treatment. While the CORT-treated adult animals showed significantly larger startle responses relative to vehicle-treated animals (*t*_(15)_ = 2.77, *P* = 0.014), smaller startle responses were found in the adolescent animals receiving CORT treatment compared to vehicle-treated animals (*t*_(15)_ = 2.21, *P* = 0.043).

#### Spatial Learning in the Morris Water Maze

**Figure 6:**
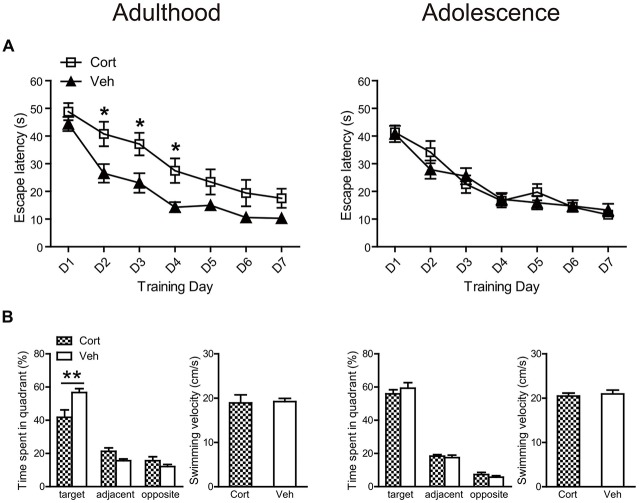
**Effects of chronic CORT treatment in adult and adolescent animals on spatial learning in the Morris water maze. (A)** Mean escape latency within each training day. **(B)** The percentage time animals spent in the target, adjacent and opposite quadrants and the mean swim speed during the probe test. For adult animals, *n* = 14 in the CORT group and *n* = 18 in the vehicle group; for adolescent animals, *n* = 19 in the CORT group and *n* = 20 in the vehicle group. *p < 0.05, **p < 0.01, compared with the vehicle group. Cort, corticosterone; Veh, vehicle.

For spatial learning, chronic CORT treatment produced deleterious effects in adult, but not adolescent animals (Figure 6A). For adult animals, repeated measures ANOVA with escape latency over seven consecutive learning days showed significant main effects of time (*F*_(6,180)_ = 45.90, *P* < 0.001) and treatment (*F*_(1,30)_ = 9.93, *P* = 0.004), with a lack of interaction (*F*_(6,180)_ = 1.19, *P* = 0.32). Analyzing the escape latency within each training day indicated that CORT-treated adult animals spent more time than vehicle-treated animals to find the platform throughout the learning period, with treatment effects approaching significance from the second to the fourth learning day (*P*s < 0.014). For adolescent animals, repeated measures ANOVA revealed a significant main effect of day (*F*_(6,222)_ = 47.71, *P* < 0.001) with a lack of main effect of treatment (*F*_(1,37)_ = 0.086, *P* = 0.77) and day × treatment interaction (*F*_(6,222)_ = 1.16, *P* = 0.33), suggesting that CORT- and vehicle-treated animals had similar spatial learning performance. Similar results were found when swimming distance was analyzed.

These results were confirmed in the following probe test (Figure 6B). Although all groups of animals spent relatively more time in the target quadrant, where the platform had been located during the learning phase, than those quadrants that were adjacent or opposite to the target one (*P*s < 0.013), only adult animals receiving CORT treatment spent significantly less time in the target quadrant relative to vehicle-treated animals (*t*_(32)_ = 21.70, *P* = 0.003). The swimming speed in the probe test was similar for CORT- and vehicle-treated groups in both ages (Figure 6B).

### Neurobiological Effects of Chronic CORT Treatment in Adult and Adolescent Animals

#### BDNF and Cell Adhesion Molecules

**Figure 7:**
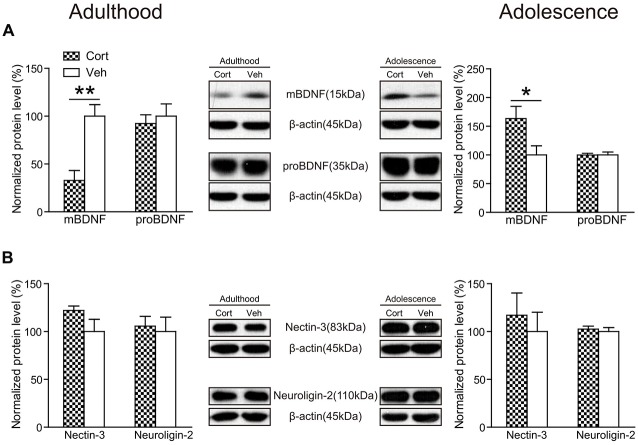
**Effects of chronic CORT treatment in adult and adolescent animals on hippomcampal BDNF and cell adhesion molecules.** Representative Western blots and relative bar graphs showing the expression levels of **(A)** mBDNF and proBDNF, **(B)** nectin-3 and neuroligin-2. *n* = 8 per group for mBDNF, proBDNF and neuroligin-2 in both ages and for nectin-3 in adolescent animals; *n* = 6 per group for nectin-3 in adult animals. *p < 0.05, **p < 0.01, compared with the vehicle group. Cort, corticosterone; Veh, vehicle.

Chronic CORT treatment was found to exert opposite effects on hippocampal mature BDNF (mBDNF) levels in the two age groups (Figure 7A). While mBDNF expression levels were significantly decreased in CORT-treated adult animals (*t*_(14)_ = 4.23, *P* = 0.001), they were significantly upregulated in CORT-treated adolescent animals (*t*_(14)_ = 2.40, *P* = 0.031). We also detected the expression levels of the precursor of BDNF (proBDNF), which has different effects from mBDNF in the regulation of neurite growth, spine formation and cell survival (Gibon et al., 2016; Orefice et al., 2016). In our study, we found no alterations of proBDNF in either age group following chronic CORT treatment (*P*s > 0.63).

Recent evidence indicates that hippocampal cell adhesion molecules, including nectin-3 and neuroligin-2, are key modulators in stress-induced behavioral alterations (Wang et al., 2013; Kohl et al., 2015). Here we examined the influence of chronic CORT treatment on the expression levels of nectin-3 and neuroligin-2 in adult and adolescent animals. The results showed, however, that neither nectin-3 nor neuroligin-2 was significantly altered by chronic CORT treatment in rat hippocampus in the adult or adolescent group (*P*s > 0.14; Figure 7B), indicating that chronic CORT treatment had negligible impact in the expression levels of these cell adhesion molecules in rat hippocampus.

#### Glutamate Receptors

**Figure 8:**
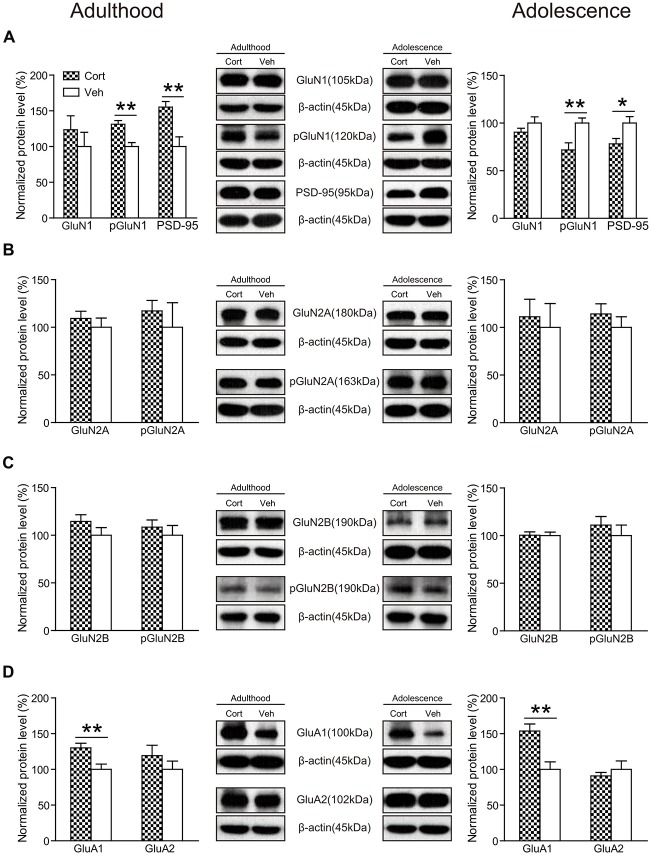
**Effects of chronic CORT treatment in adult and adolescent animals on the expression levels of glutamate receptors in rat hippocampus.** Representative Western blots and relative bar graphs showing the expression levels of **(A)** GluN1, its phosphorylated form and PSD-95, **(B)** GluN2 and its phosphorylated form, **(C)** GluN3 and its phosphorylated form, **(D)** AMPA receptor subunits. *n* = 8 per group for GluN1, phosphorylation of GluN1 (pGluN1), PSD-95 in both ages, for GluN2B, pGluN2B, GluA1 in adult animals and for GluN2A, GluN2B, GluA1 and GluA2 in adolescent animals; *n* = 7 per group for GluA2 in adult animals; *n* = 6 per group for pGluN2A in adult animals. For GluN2A in adult animals, *n* = 8 in the CORT group and *n* = 6 in the vehicle group; for pGluN2A and pGluN2B in adolescent animals, *n* = 8 in the CORT group and *n* = 7 in the vehicle group. *p < 0.05, **p < 0.01, compared with the vehicle group. Cort, corticosterone; Veh, vehicle.

As shown in Figure 8A, chronic CORT treatment did not significantly affect the expression levels of GluN1 in either adult or adolescent animals (*P*s > 0.24). However, the phosphorylation of GluN1 (pGluN1) was altered in an age-dependent manner. Compared to vehicle-treated animals, this protein was significantly upregulated in CORT-treated adult animals (*t*_(14)_ = 4.08, *P* = 0.001), but downregulated in CORT-treated adolescent animals (*t*_(14)_ = 3.07, *P* = 0.008). Similar changes were observed for the postsynaptic density protein 95 (PSD-95) in that CORT treatment induced a significant increase of PSD-95 levels in adult animals (*t*_(14)_ = 3.52, *P* = 0.003), but a decrease in adolescent animals (*t*_(14)_ = 2.49, *P* = 0.026). The expression levels of GluN2A, GluN2B as well as their phosphorylation were unchanged in either age group (*P*s > 0.20; Figures 8B,C).

We also measured the expression levels of AMPA receptors and found that chronic CORT treatment significantly upregulated the expression levels of the GluA1 subunit in both adolescent (*t*_(14)_ = 3.71, *P* = 0.002) and adult groups (*t*_(14)_ = 3.03, *P* = 0.009). No significant changes were observed for the GluA2 subunit in either age group (*P*s > 0.32; Figure 8D).

The expression levels of GAD67 were measured to examine the potential changes associated with inhibitory neurotransmission following chronic CORT treatment. No significant alterations were noted in either age group (*P*s > 0.64).

## Discussion

The present study showed that chronic CORT treatment produced differential behavioral and neurobiological effects on adolescent and adult animals, despite of similar physical and neuroendocrine effects. Behaviorally, adult animals exposed to CORT exhibited decreased preference for sucrose solution (indicative of anhedonia), increased anxiety levels, and impaired cognitive performance compared to vehicle-treated animals. These are consistent with previous findings supporting chronic CORT treatment during adulthood as a reliable animal model mimicking some of the key symptoms of depression (Sterner and Kalynchuk, 2010). Adolescent animals receiving the same treatment, however, showed an increase in sucrose intake and preference, reduced startle amplitude during the familiarization period (indicative of decreased anxiety levels) and PPI enhancement at a low prepulse threshold. Paralleling these age-dependent behavioral changes, the expression levels of BDNF, the phosphorylation of the obligatory subunit of NMDA receptors, GluN1, and PSD-95 in rat hippocampus were altered in opposite directions in the two age groups. Taken together, these findings suggest that prolonged glucocorticoid exposure in adolescent individuals does not produce typical stress-like adverse effects as shown in adults, but rather results in abnormal reward and emotion processing that could link to psychiatric diseases such as bipolar disorder.

Several previous studies have investigated the effects of chronic CORT exposure during adolescence. Most of them administered CORT via drinking water with various doses (Xu et al., 2011; Klug et al., 2012; Torregrossa et al., 2012; Buret and van den Buuse, 2014; Den et al., 2014; Kaplowitz et al., 2016) except that one study (Waters and McCormick, 2011) used daily injection of CORT at the same dose in our study (i.e., 40 mg/kg), but with shorter treatment period (2 weeks in Waters and McCormick, 2011 vs. 3 weeks in our study). The majority of these studies examined behavioral effects in one particular domain, such as fear extinction (Den et al., 2014), impulsivity (Torregrossa et al., 2012). In this context, the novelty of our study lies in providing a systematic evaluation of stress-related behaviors, such as depression-like, anxiety-like and cognitive performance, and more importantly, including adulthood as a control to highlight the age-specific responses to repeated CORT exposure.

Chronic CORT treatment produced similar physical and neuroendocrine effects in both adult and adolescent animals, as shown in reduction in body weight, plasma CORT levels, relative weights of adrenal glands and hippocampal MR expression. This indicates that the treatment was equally effective in attenuating the endogenous HPA-axis functioning in both age groups (Xu et al., 2011; Demuyser et al., 2016), and that age-dependent behavioral and neurobiological effects following this treatment may not be attributed to these physical and neuroendocrine effects.

The sucrose preference test is widely used to measure hedonic and reward responses (Willner, 2005; McCormick and Green, 2013). Reduced consumption and preference of the sucrose solution is considered anhedonia, one core symptom of depression, and has been consistently found in animal models of depression, such as chronic CORT treatment in adult animals (Sterner and Kalynchuk, 2010; Yau et al., 2014; Weng et al., 2016). Previous studies investigating the effects of chronic adolescent stress on this test report controversial results, with anhedonia found in adolescent animals exposed to chronic restraint stress (Eiland et al., 2012), not in those exposed to chronic mild stress (Pohl et al., 2007; Toth et al., 2008). Here we found that CORT-related adolescent animals did not exhibit anhedonia as adult animals did; instead they showed increased sucrose consumption and preference that persisted 1 week after the last injection. This pattern was more prominent at a low-concentration sucrose solution, which may indicate that the brain’s reward circuitry is altered in such a way that the ability to perceive and respond to small rewards is augmented following chronic CORT treatment. It is also not unlikely that the enhanced pursue of natural rewards may render adolescents at a higher risk of drug abuse and addiction (Levine et al., 2003).

Anxiety-related behaviors in rodents can be assessed in various paradigms (McCormick and Green, 2013). We used two tasks that have been considered as expression of anxiety/fear, including the open field (Prut and Belzung, 2003) and startle responses (Garrick et al., 2001). While the open field reflects a conflict situation between animals’ fear of open space and novelty-induced exploratory drive, startle responses are reflexive reactions to an intense and surprising stimulus and may correspond to defensive flight in the multidimensional structure of anxiety-related behaviors (Lopez-Aumatell et al., 2008). We found that in both measures CORT-treated adult animals exhibited increased anxiety levels, a finding commonly observed in previous studies with exogenous CORT administration or chronic stress (Willner, 2005; Sterner and Kalynchuk, 2010). In adolescent animals, although there were studies reporting enhanced anxiety in adult animals previously exposed to chronic stress during adolescence (McCormick and Green, 2013), studies investigating the immediate effects of chronic adolescent stress, as typically examined in adulthood, did not report increased anxiety levels in tests including elevated plus maze and open field following various physical and psychological stress paradigms (Leussis and Andersen, 2008; McCormick et al., 2008; Eiland et al., 2012; Yuen et al., 2012). Our study extends this finding to the paradigm of chronic CORT treatment, showing that this paradigm did not alter animals’ free exploration in the open field. Nevertheless, we found that repeated CORT treatment reduced the acoustic startle response in the familiarization phase of the PPI test. It is worth mentioning that a reduction of startle responses has been reported in adolescent animals housed in isolation (Jones et al., 2011). These results indicate that chronic stress or exposure to CORT may render adolescent individuals less defensive in face of unexpected stimuli. Future studies are needed to evaluate whether this effect in adolescent animals can be generalized to other contexts.

It has been well-established that chronic stress during adulthood adopting various paradigms impairs hippocampus-dependent cognitive performance (Willner, 2005; Sterner and Kalynchuk, 2010). However, relatively mild effects or no deleterious effects were observed following chronic stress in adolescence (Green and McCormick, 2013). We found that this age-dependent pattern still holds in the paradigm of chronic CORT treatment, with the spatial learning performance impaired in adult animals, but not in adolescent animals. PPI is an operational measure of sensorimotor gating, reflecting a neurocognitive process of filtering responses to incoming external information (Braff and Geyer, 1990). We found that while adult animals receiving chronic CORT treatment exhibited a nonsignificant reduction of PPI levels, the CORT-treated adolescent animals showed significantly increased PPI levels when the prepulse intensity was weak. This effect cannot be explained by greater prepulse-elicited reactivity. Moreover, no PPI improvement was detected with higher prepulse intensities probably due to a ceiling effect. Indeed, *post hoc* between-prepulse comparisons showed that the PPI levels at the 12 dB prepulse were not enhanced in CORT-treated adolescent animals compared to those at the lower prepulse intensities (*P*s > 0.23), whereas in vehicle-treated animals PPI increases were significant (*P*s < 0.002). Future investigation is warranted to examine the generalization of the improved sensorimotor gating in other sensory modalities and to uncover the underlying mechanisms. Nonetheless, this result echoes our findings of increased basal sensitivity to rewards at a low concentration of the sucrose solution and of a less defensive state. These alterations together may indicate behavioral arousal in adolescent animals following chronic CORT treatment.

At the neurobiological level, we first found that hippocampal BDNF levels were modulated in an age-dependent manner following chronic CORT treatment. Consistent with previous studies of chronic CORT treatment (Dwivedi et al., 2006; Jacobsen and Mørk, 2006), we observed significant reduction in the expression levels of BDNF in CORT-treated adult animals. Intriguingly, we found that hippocampal BDNF levels were significantly increased in CORT-treated adolescent animals compared to vehicle-treated animals. Upregulation of hippocampal BDNF levels have also been found in adolescent animals after chronic mild stress (Toth et al., 2008) and social defeat (Coppens et al., 2011). These results suggest that increasing BDNF levels is a general age-specific consequence when the developing teenage brain is exposed to chronic stress or prolonged CORT administration.

We then investigated the effects of chronic CORT treatment on glutamate neurotransmission. The GluA1 subunit of the AMPA receptor was significantly upregulated following the treatment in both adolescent and adult animals. Age-dependent effects, however, were observed in the pGluN1, the obligatory subunit of the NMDA receptors, and PSD-95, a postsynaptic protein that stably co-localizes with NMDA receptors. In adult animals, the pGluN1 and PSD-95 were significantly upregulated by CORT. Similar upregulation of NMDA receptor subunits in rat hippocampus have been found after chronic mild stress (Calabrese et al., 2012). Note that one previous study found that 7-day CORT pellet exposure in adult, male C57BL/6J mice did not alter GluN1 and GluN2A gene expression, but significantly decreased GluN2B and GluN2C levels in the hippocampus (Hodes et al., 2012). This is not surprising when considering that chronic CORT treatment in Hodes et al. (2012) study and our study had different neuroendocrine effects. The former reported increased plasma CORT levels and decreased expression levels of both MR and GR in the hippocampus, whereas we found decreased plasma CORT levels and a reduction of MR expression only. Such inconsistency may result from differences in treatment approaches, duration, doses and animal strains. In comparison, chronic CORT treatment decreased the pGluN1 and PSD-95 expression in adolescent animals. The suppression of glutamate receptor expression and function has been documented in adolescent rats following 7-day chronic stress in prefrontal cortex, although not in hippocampus (Yuen et al., 2012).

The differential behavioral and neurobiological effects of chronic CORT treatment in adult and adolescent animals indicate that repeated exposure to CORT evokes distinct mechanisms in the brain in the two age periods. For adult animals, our findings of increased NMDA receptor functions and previous observations of increased glutamate release and decreased glutamate uptake in the hippocampus following chronic stress (Fontella et al., 2004; de Vasconcellos-Bittencourt et al., 2011) together suggest a state of enhanced glutamate activity after chronic stress or exposure to CORT (Popoli et al., 2012). The altered glutamate neurotransmission could lead to a wide range of deleterious consequences, including dendritic atrophy, reduced neurogenesis and impaired synaptic plasticity (Sterner and Kalynchuk, 2010).

Different from adulthood, adolescence is a critical neurodevelopmental period (Spear, 2000). By comparing the behavioral and neurobiological measurements in adolescent and adult controls, we observed several developmental changes (Supplementary Figures S2–S4). For instance, from adolescence to adulthood, animals became less active in the locomotor activity box, showed increased preference to the 0.2% sucrose solution and increased sensorimotor gating functions (Supplementary Figure S2); hippocampal expression levels of BDNF, GluN1, pGluN1 and PSD-95 were reduced, whereas the proBDNF levels were upregulated (Supplementary Figure S3). Considering the CORT-induced adolescence-specific alterations in this context, we speculate that chronic CORT treatment may render adolescent animals more adult-like to some extent, except for the BDNF levels and the startle response during the familiarization phase. It is thus possible that repeated exposure to CORT during adolescence may promote the neurodevelopmental processes, such as synaptic pruning mediated by NMDA receptor-dependent long-term depression (Selemon, 2013). The CORT-induced BDNF upregulation in adolescent animals could either serve as a compensatory mechanism to the potential overpruning or accelerated pruning (Tyler and Pozzo-Miller, 2003) or may reflect the modulation of other aspects of adolescent network remodeling, such as axon pruning (Singh et al., 2008). All these possibilities warrant further investigation.

It should be noted that there are a number of limitations to our study. First, the age-dependent effects we observed with chronic CORT treatment do not necessarily ensure that similar consequences occur after chronic stress exposure. After all, the pharmacological model we adopted here does not simulate real-life stressors to which animals can develop individual coping strategies; it should be better considered as a model investigating the involvement of CORT in stress-related pathophysiological mechanisms. Second, protein levels in our study were examined in the entire hippocampus. As there are functional subdivisions in the hippocampus (Fanselow and Dong, 2011) that are involved in cognition and emotion, respectively, future studies are needed to identify CORT-induced neurobiological changes in each subregion and to examine how these changes relate to behaviors. Finally, the behavioral phenotype of CORT-treated adolescent animals in our study were immediate effects of chronic CORT treatment except that the increase in sucrose intake and preference persisted 1 week after treatment cessation. The long-term effects of such treatment still await investigation. It is possible that the behavioral changes with anxiety and PPI we observed may disappear when these animals grow to adulthood (Klug et al., 2012). It is also not unlikely that chronic CORT treatment during adolescence may result in some potential deleterious effects that may become evident when the neurodevelopmental processes come to an end (Torregrossa et al., 2012; Green and McCormick, 2013).

To conclude, our study reveals the striking distinction of behavioral effects of chronic CORT treatment in adult and adolescent animals. While adult animals exhibited a depression-like phenotype following the treatment, adolescent individuals presented behavioral changes in opposite directions, including increased sensitivity to rewards, decreased anxiety levels and enhanced PPI levels. We further found that these age-dependent behavioral effects may be mediated by differential alterations of the NMDA receptors and BDNF levels in the hippocampus. Together these results highlight the interaction between stress hormones and the neurodevelopmental processes during adolescence, probably involving NMDA receptors and BDNF expression, and invite future studies to probe into the underlying mechanisms of stress responses in the adolescent brain.

## Author Contributions

Y-AS and TS designed research; JL, XX, YL and XLiu performed research; JL, XX, XLiu and XLiao analyzed data; JL, XX, Y-AS and TS wrote the manuscript.

## Funding

This work was supported by the National Natural Science Foundation of China (grant No. 81401129, 81571321 and 81571312), the National Key Basic Research Program of China (973 program, No. 2015CB856400) and the Research Fund for the Doctoral Program of Higher Education of China (grant No. 20130001120118).

## Conflict of Interest Statement

The authors declare that the research was conducted in the absence of any commercial or financial relationships that could be construed as a potential conflict of interest.

## References

[B1] Bingham B. McFadden K. Zhang X. Bhatnagar S. Beck S. Valentino R. (2011). Early adolescence as a critical window during which social stress distinctly alters behavior and brain norepinephrine activity. Neuropsychopharmacology 36, 896–909. 10.1038/npp.2010.229 21178981 PMC3055730

[B2] Braff D. L. Geyer M. A. (1990). Sensorimotor gating and schizophrenia: Human and animal model studies. Arch. Gen. Psychiatry 47, 181–188. 10.1001/archpsyc.1990.01810140081011 2405807

[B3] Buret L. van den Buuse M. (2014). Corticosterone treatment during adolescence induces down-regulation of reelin and NMDA receptor subunit GLUN2C expression only in male mice: implications for schizophrenia. Int. J. Neuropsychopharmacol. 17, 1221–1232. 10.1017/s1461145714000121 24556017

[B4] Calabrese F. Guidotti G. Molteni R. Racagni G. Mancini M. Riva M. A. (2012). Stress-induced changes of hippocampal NMDA receptors: modulation by duloxetine treatment. PLoS One 7:e37916. 10.1371/journal.pone.0037916 22666412 PMC3362535

[B5] Coppens C. M. Siripornmongcolchai T. Wibrand K. Alme M. N. Buwalda B. de Boer S. F.. (2011). Social defeat during adolescence and adulthood differentially induce bdnf-regulated immediate early genes. Front. Behav. Neurosci. 5:72. 10.3389/fnbeh.2011.00072 22065953 PMC3206404

[B7] Demuyser T. Bentea E. Deneyer L. Albertini G. Massie A. Smolders I. (2016). Disruption of the HPA-axis through corticosterone-release pellets induces robust depressive-like behavior and reduced BDNF levels in mice. Neurosci. Lett. 626, 119–125. 10.1016/j.neulet.2016.05.026 27208833

[B8] Den M. L. Altmann S. R. Richardson R. (2014). A comparison of the short- and long-term effects of corticosterone exposure on extinction in adolescence versus adulthood. Behav. Neurosci. 128, 722–735. 10.1037/bne0000022 25314660

[B9] Doremus-Fitzwater T. L. Varlinskaya E. I. Spear L. P. (2009). Social and non-social anxiety in adolescent and adult rats after repeated restraint. Physiol. Behav. 97, 484–494. 10.1016/j.physbeh.2009.03.025 19345235 PMC2693722

[B10] Duman R. S. Monteggia L. M. (2006). A neurotrophic model for stress-related mood disorders. Biol. Psychiatry 59, 1116–1127. 10.1016/j.biopsych.2006.02.013 16631126

[B11] Dwivedi Y. Rizavi H. S. Pandey G. N. (2006). Antidepressants reverse corticosterone-mediated decrease in brain-derived neurotrophic factor expression: differential regulation of specific exons by antidepressants and corticosterone. Neuroscience 139, 1017–1029. 10.1016/j.neuroscience.2005.12.058 16500030 PMC1513636

[B12] Eiland L. Ramroop J. Hill M. N. Manley J. McEwen B. S. (2012). Chronic juvenile stress produces corticolimbic dendritic architectural remodeling and modulates emotional behavior in male and female rats. Psychoneuroendocrinology 37, 39–47. 10.1016/j.psyneuen.2011.04.015 21658845 PMC3181388

[B13] Fanselow M. S. Dong H.-W. (2011). Are the dorsal and ventral hippocampus functionally distinct structures? Neuron 65, 7–19. 10.1016/j.neuron.2009.11.031 20152109 PMC2822727

[B14] Fontella F. Vendite D. A. Tabajara A. Porciúncula L. da Silva Torres I. Jardim F.. (2004). Repeated restraint stress alters hippocampal glutamate uptake and release in the rat. Neurochem. Res. 29, 1703–1709. 10.1023/b:nere.0000035805.46592.6c 15453265

[B15] Garrick T. Morrow N. Shalev A. Y. Eth S. (2001). Stress-induced enhancement of auditory startle: an animal model of posttraumatic stress disorder. Psychiatry 64, 346–354. 10.1521/psyc.64.4.346.18600 11822211

[B660] Gearhart D. A. Middlemore M. L. Terry A. V. (2006). ELISA methods to measure cholinergic markers and nerve growth factor receptors in cortex, hippocampus, prefrontal cortex, and basal forebrain from rat brain. J. Neurosci. Methods 150, 159–173. 10.1016/j.jneumeth.2005.06.009 16085318

[B16] Gibon J. Barker P. A. Séguéla P. (2016). Opposing presynaptic roles of BDNF and ProBDNF in the regulation of persistent activity in the entorhinal cortex. Mol. Brain 9:23. 10.1186/s13041-016-0203-9 26932787 PMC4774087

[B17] Graaf C. D. E. Zandstra E. H. (1999). Sweetness intensity and pleasantness in children, adolescents and adults. Physiol. Behav. 67, 513–520. 10.1016/s0031-9384(99)00090-6 10549887

[B18] Green M. R. McCormick C. M. (2013). Effects of stressors in adolescence on learning and memory in rodent models. Horm. Behav. 64, 364–379. 10.1016/j.yhbeh.2012.09.012 23998678

[B19] Hammen C. L. (2005). Stress and depression. Annu. Rev. Clin. Psychol. 1, 293–319. 10.1146/annurev.clinpsy.1.102803.143938 17716090

[B20] Hashimoto K. (2010). Brain-derived neurotrophic factor as a biomarker for mood disorders: an historical overview and future directions. Psychiatry Clin. Neurosci. 64, 341–357. 10.1111/j.1440-1819.2010.02113.x 20653908

[B21] Hodes G. E. Brookshire B. R. Hill-Smith T. E. Teegarden S. L. Berton O. Lucki I. (2012). Strain differences in the effects of chronic corticosterone exposure in the hippocampus. Neuroscience 222, 269–280. 10.1016/j.neuroscience.2012.06.017 22735575 PMC3587173

[B22] Jacobsen J. P. R. Mørk A. (2006). Chronic corticosterone decreases brain-derived neurotrophic factor (BDNF) mRNA and protein in the hippocampus, but not in the frontal cortex, of the rat. Brain Res. 1110, 221–225. 10.1016/j.brainres.2006.06.077 16876769

[B23] Joëls M. Baram T. Z. (2009). The neuro-symphony of stress. Nat. Rev. Neurosci. 10, 459–466. 10.1038/nrn2632 19339973 PMC2844123

[B24] Jones C. A. Brown A. M. Auer D. P. Fone K. C. F. (2011). The mGluR2/3 agonist LY379268 reverses post-weaning social isolation-induced recognition memory deficits in the rat. Psychopharmacology (Berl) 214, 269–283. 10.1007/s00213-010-1931-7 20607219

[B25] Kaplowitz E. T. Savenkova M. Karatsoreos I. N. Romeo R. D. (2016). Somatic and neuroendocrine changes in response to chronic corticosterone exposure during adolescence in male and female rats. J. Neuroendocrinol. 28:12336. 10.1111/jne.12336 26568535

[B26] Klug M. Hill R. A. Choy K. H. C. Kyrios M. Hannan A. J. van den Buuse M. (2012). Long-term behavioral and NMDA receptor effects of young-adult corticosterone treatment in BDNF heterozygous mice. Neurobiol. Dis. 46, 722–731. 10.1016/j.nbd.2012.03.015 22426399

[B27] Kohl C. Wang X. D. Grosse J. Fournier C. Harbich D. Westerholz S.. (2015). Hippocampal neuroligin-2 links early-life stress with impaired social recognition and increased aggression in adult mice. Psychoneuroendocrinology 55, 128–143. 10.1016/j.psyneuen.2015.02.016 25765754

[B28] Leussis M. P. Andersen S. L. (2008). Is adolescence a sensitive period for depression? Behavioral and neuroanatomical findings from a social stress model. Synapse 62, 22–30. 10.1002/syn.20462 17957735

[B29] Levine A. S. Kotz C. M. Gosnell B. A. (2003). Sugars: hedonic aspects, neuroregulation and energy balance. Am. J. Clin. Nutr. 78, 834S–842S. 14522747 10.1093/ajcn/78.4.834S

[B30] Li J. T. Feng Y. Su Y. A. Wang X. D. Si T. M. (2013). Enhanced interaction among ErbB4, PSD-95 and NMDAR by chronic MK-801 treatment is associated with behavioral abnormalities. Pharmacol. Biochem. Behav. 108, 44–53. 10.1016/j.pbb.2013.04.008 23603030

[B31] Lopez-Aumatell R. Guitart-Masip M. Vicens-Costa E. Gimenez-Llort L. Valdar W. Johannesson M.. (2008). Fearfulness in a large N/Nih genetically heterogeneous rat stock: differential profiles of timidity and defensive flight in males and females. Behav. Brain Res. 188, 41–55. 10.1016/j.bbr.2007.10.015 18079010

[B32] Lyons D. M. Parker K. J. Schatzberg A. F. (2010). Animal models of early life stress: implications for understanding resilience. Dev. Psychobiol. 52, 616–624. 10.1002/dev.20500 20957724 PMC6716163

[B33] McCormick C. M. Green M. R. (2013). From the stressed adolescent to the anxious and depressed adult: investigations in rodent models. Neuroscience 249, 242–257. 10.1016/j.neuroscience.2012.08.063 22967838

[B34] McCormick C. M. Smith C. Mathews I. Z. (2008). Effects of chronic social stress in adolescence on anxiety and neuroendocrine response to mild stress in male and female rats. Behav. Brain Res. 187, 228–238. 10.1016/j.bbr.2007.09.005 17945360

[B35] McEwen B. S. (2007). Physiology and neurobiology of stress and adaptation: central role of the brain. Physiol. Rev. 87, 873–904. 10.1152/physrev.00041.2006 17615391

[B36] Morrissey M. D. Mathews I. Z. McCormick C. M. (2011). Enduring deficits in contextual and auditory fear conditioning after adolescent, not adult, social instability stress in male rats. Neurobiol. Learn. Mem. 95, 46–56. 10.1016/j.nlm.2010.10.007 20970512

[B37] O’Donnell P. (2011). Adolescent onset of cortical disinhibition in schizophrenia: insights from animal models. Schizophr. Bull. 37, 484–492. 10.1093/schbul/sbr028 21505115 PMC3080677

[B38] Orefice L. L. Shih C. Xu H. Waterhouse E. G. Xu B. (2016). Molecular and cellular neuroscience control of spine maturation and pruning through proBDNF synthesized and released in dendrites. Mol. Cell. Neurosci. 71, 66–79. 10.1016/j.mcn.2015.12.010 26705735 PMC4761458

[B39] Pohl J. Olmstead M. C. Wynne-Edwards K. E. Harkness K. Menard J. L. (2007). Repeated exposure to stress across the childhood-adolescent period alters rats’ anxiety- and depression-like behaviors in adulthood: the importance of stressor type and gender. Behav. Neurosci. 121, 462–474. 10.1037/0735-7044.121.3.462 17592937

[B40] Popoli M. Yan Z. McEwen B. S. Sanacora G. (2012). The stressed synapse: the impact of stress and glucocorticoids on glutamate transmission. Nat. Rev. Neurosci. 13, 22–37. 10.1038/nrn3138 22127301 PMC3645314

[B41] Prut L. Belzung C. (2003). The open field as a paradigm to measure the effects of drugs on anxiety-like behaviors: a review. Eur. J. Pharmacol. 463, 3–33. 10.1016/s0014-2999(03)01272-x 12600700

[B42] Regev L. Tsoory M. Gil S. Chen A. (2012). Site-specific genetic manipulation of amygdala corticotropin-releasing factor reveals its imperative role in mediating behavioral response to challenge. Biol. Psychiatry 71, 317–326. 10.1016/j.biopsych.2011.05.036 21783178

[B43] Ricon T. Toth E. Leshem M. Braun K. Richter-Levin G. (2012). Unpredictable chronic stress in juvenile or adult rats has opposite effects, respectively, promoting and impairing resilience. Stress 15, 11–20. 10.3109/10253890.2011.572207 21682654

[B44] Romeo R. D. (2013). The teenage brain: the stress response and the adolescent brain. Curr. Dir. Psychol. Sci. 22, 140–145. 10.1177/0963721413475445 25541572 PMC4274618

[B45] Romeo R. D. (2015). Perspectives on stress resilience and adolescent neurobehavioral function. Neurobiol. Stress 1, 128–133. 10.1016/j.ynstr.2014.11.001 27589663 PMC4721430

[B46] Sapolsky R. M. (2015). Stress and the brain: individual variability and the inverted-U. Nat. Neurosci. 18, 1344–1346. 10.1038/nn.4109 26404708

[B47] Selemon L. D. (2013). A role for synaptic plasticity in the adolescent development of executive function. Transl. Psychiatry 3:e238. 10.1038/tp.2013.7 23462989 PMC3625918

[B48] Singh K. K. Park K. J. Hong E. J. Kramer B. M. Greenberg M. E. Kaplan D. R.. (2008). Developmental axon pruning mediated by BDNF-p75NTR-dependent axon degeneration. Nat. Neurosci. 11, 649–658. 10.1038/nn.2114 18382462

[B49] Spear L. P. (2000). The adolescent brain and age-related behavioral manifestations. Neurosci. Biobehav. Rev. 24, 417–463. 10.1016/s0149-7634(00)00014-2 10817843

[B50] Sterner E. Y. Kalynchuk L. E. (2010). Behavioral and neurobiological consequences of prolonged glucocorticoid exposure in rats: relevance to depression. Prog. Neuropsychopharmacol. Biol. Psychiatry 34, 777–790. 10.1016/j.pnpbp.2010.03.005 20226827

[B51] Torregrossa M. M. Xie M. Taylor J. R. (2012). Chronic corticosterone exposure during adolescence reduces impulsive action but increases impulsive choice and sensitivity to yohimbine in male Sprague-Dawley rats. Neuropsychopharmacology 37, 1656–1670. 10.1038/npp.2012.11 22334120 PMC3358734

[B52] Toth E. Gersner R. Wilf-Yarkoni A. Raizel H. Dar D. E. Richter-Levin G.. (2008). Age-dependent effects of chronic stress on brain plasticity and depressive behavior. J. Neurochem. 107, 522–532. 10.1111/j.1471-4159.2008.05642.x 18752645

[B53] Tyler W. J. Pozzo-Miller L. (2003). Miniature synaptic transmission and BDNF modulate dendritic spine growth and form in rat CA1 neurones. J. Physiol. 553, 497–509. 10.1113/jphysiol.2003.052639 14500767 PMC2343578

[B54] Varese F. Smeets F. Drukker M. Lieverse R. Lataster T. Viechtbauer W.. (2012). Childhood adversities increase the risk of psychosis: a meta-analysis of patient-control, prospective-and cross-sectional cohort studies. Schizophr. Bull. 38, 661–671. 10.1093/schbul/sbs050 22461484 PMC3406538

[B6] de Vasconcellos-Bittencourt A. P. Vendite D. A. Nassif M. Crema L. M. Frozza R. Thomazi A. P.. (2011). Chronic stress and lithium treatments alter hippocampal glutamate uptake and release in the rat and potentiate necrotic cellular death after oxygen and glucose deprivation. Neurochem. Res. 35, 793–800. 10.1007/s11064-011-0404-7 21253855

[B55] Vásquez C. E. Riener R. Reynolds E. Britton G. B. (2014). NMDA receptor dysregulation in chronic state: a possible mechanism underlying depression with BDNF downregulation. Neurochem. Int. 79, 88–97. 10.1016/j.neuint.2014.09.007 25277075

[B56] Vorhees C. V. Williams M. T. (2006). Morris water maze: procedures for assessing spatial and related forms of learning and memory. Nat. Protoc. 1, 848–858. 10.1038/nprot.2006.116 17406317 PMC2895266

[B57] Wang X.-D. Su Y.-A. Wagner K. V. Avrabos C. Scharf S. H. Hartmann J.. (2013). Nectin-3 links CRHR1 signaling to stress-induced memory deficits and spine loss. Nat. Neurosci. 16, 706–713. 10.1038/nn.3395 23644483

[B58] Waters P. McCormick C. M. (2011). Caveats of chronic exogenous corticosterone treatments in adolescent rats and effects on anxiety-like and depressive behavior and hypothalamic-pituitary-adrenal (HPA) axis function. Biol. Mood Anxiety Disord. 1:4. 10.1186/2045-5380-1-4 22738136 PMC3377168

[B59] Weng L. Guo X. Li Y. Yang X. Han Y. (2016). Apigenin reverses depression-like behavior induced by chronic corticosterone treatment in mice. Eur. J. Pharmacol. 774, 50–54. 10.1016/j.ejphar.2016.01.015 26826594

[B60] Willner P. (2005). Chronic mild stress (CMS) revisited: consistency and behavioural- neurobiological concordance in the effects of CMS. Neuropsychobiology 52, 90–110. 10.1159/000087097 16037678

[B61] Xu Z. Zhang Y. Hou B. Gao Y. Wu Y. Zhang C. (2011). Chronic corticosterone administration from adolescence through early adulthood attenuates depression-like behaviors in mice. J. Affect. Disord. 131, 128–135. 10.1016/j.jad.2010.11.005 21122919

[B62] Yau S. Y. Li A. Zhang E. D. Christie B. R. Xu A. Lee T. M. C.. (2014). Sustained running in rats administered corticosterone prevents the development of depressive behaviors and enhances hippocampal neurogenesis and synaptic plasticity without increasing neurotrophic factor levels. Cell Transplant. 23, 481–492. 10.3727/096368914X678490 24816445

[B63] Yee B. K. Feldon J. (2009). Distinct forms of prepulse inhibition disruption distinguishable by the associated changes in prepulse-elicited reaction. Behav. Brain Res. 204, 387–395. 10.1016/j.bbr.2008.11.049 19114060

[B64] Yuen E. Y. Wei J. Liu W. Zhong P. Li X. Yan Z. (2012). Repeated stress causes cognitive impairment by suppressing glutamate receptor expression and function in prefrontal cortex. Neuron 73, 962–977. 10.1016/j.neuron.2011.12.033 22405206 PMC3302010

[B65] Zanos P. Moaddel R. Morris P. J. Georgiou P. Fischell J. Elmer G. I.. (2016). NMDAR inhibition-independent antidepressant actions of ketamine metabolites. Nature 533, 481–486. 10.1038/nature17998 27144355 PMC4922311

[B66] Zhang F. Yuan S. Shao F. Wang W. (2016). Adolescent social defeat induced alterations in social behavior and cognitive flexibility in adult mice: effects of developmental stage and social condition. Front. Behav. Neurosci. 10:149. 10.3389/fnbeh.2016.00149 27489540 PMC4951521

