# Supplementary material for: Differential Behavioral and Neurobiological Effects of Chronic Corticosterone Treatment in Adolescent and Adult Rats
Source: Front Mol Neurosci. 2017 Feb 2;10:25. doi: 10.3389/fnmol.2017.00025 (PMC5288376; doi:10.3389/fnmol.2017.00025)
Supplement: Supplementary file 1 [file DataSheet_1.docx]

Supplementary materials

**Differential behavioral and neurobiological effects of chronic corticosterone treatment in adolescent and adult rats**

Jitao Li, Xiaomeng Xie, Youhong Li, Xiao Liu, Xuemei Liao, Yun’ai Su, Tianmei Si

**Overview**

Figure S1. Specificity of the mineralocorticoid receptor (MR) antibody.

Figure S2. Age effects of behavioral tasks in control animals.

Figure S3. Age effects of BDNF and CORT-affected glutamate receptors in control animals.

Figure S4. Age effects of neuroendocrine measures in control animals.


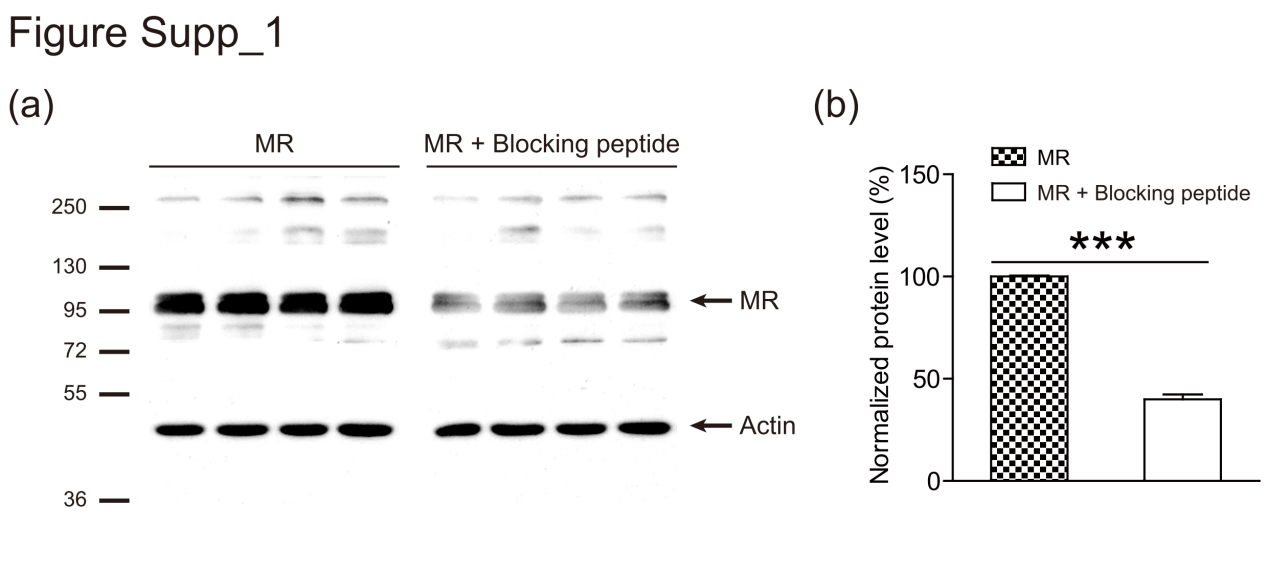


Figure S1. Specificity of the mineralocorticoid receptor (MR) antibody.

(a) The western blot of MR without and with a blocking peptide are shown respectively (*n* = 4 per group).

(b) The expression levels of both MR bands were significantly reduced by the blocking peptide (*t (3)* = 21.70, *P* < 0.001), confirming their specificity to the MR antibody. *** *P* < 0.001.


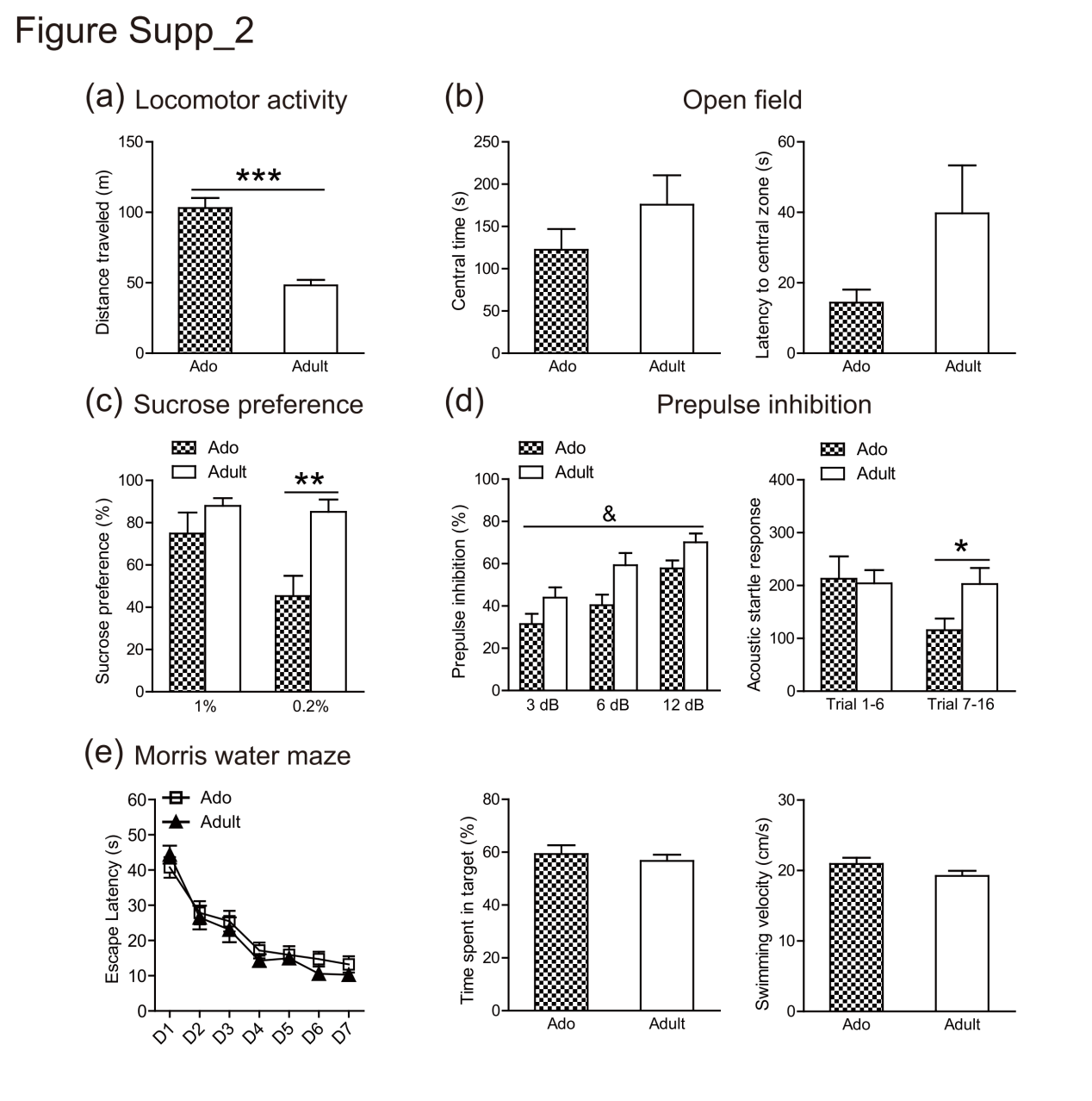


Figure S2. Age effects of behavioral tasks in control animals.

(a) Adolescent animals traveled significantly longer distance than adults (*t(18)* = 6.78, *P* < 0.001), indicative of a more active state in adolescent individuals. *n* = 10 per group.

(b) Adolescent animals showed comparable anxiety levels with adults in the open field. *n* = 10 per group.

(c) Adolescent animals showed similar preference to the 1% sucrose solution with adults, but exhibited reduced preference to the 0.2% sucrose solution (*t(16)* = -3.55, *P* = 0.003). *n* = 10 per group for the 1% sucrose solution and *n* = 9 per group for the 0.2% sucrose solution.

(d) Adolescent animals showed decreased PPI and startle responses during the test phase. Repeated measures ANOVA reveal significant main effects of age (*F (1, 16)* = 6.09, *P* = 0.025) and prepulse intensity (*F (2, 32)* = 44.33, *P* < 0.001), with a lack of age × prepulse interaction. A reduced startle response during the test phase was found in adolescent animals compared to adults (*t(16)* = -2.33, *P* = 0.033). *n* = 9 per group.

(e) No age differences were found in the spatial learning performance in the Morris water maze. *n* = 20 in the adolescent group and *n* = 18 in the adult group.

**P* < 0.05, ***P* < 0.01, *** *P* < 0.001, compared with the adult group. ^&^*P* < 0.05, indicating the significance of the main effect of age. Ado, adolescence; Adult, adulthood.


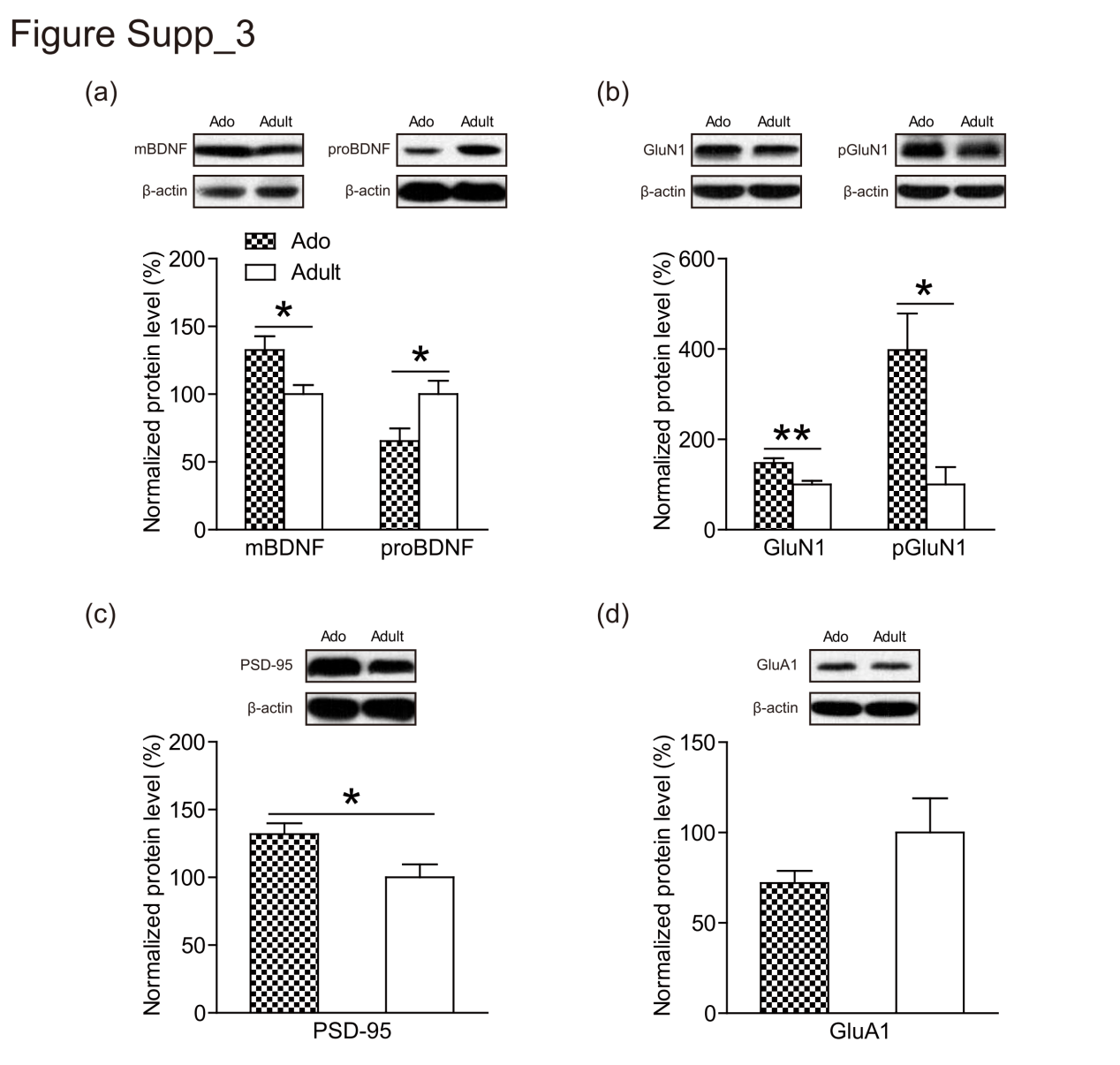


Figure S3. Age effects of BDNF and CORT-affected glutamate receptors in control animals.

(a) From adolescence to adulthood, mBDNF was significantly decreased (*t(10)* = -2.81, *P* = 0.019) whereas proBDNF was significantly increased (*t(9)* = -2.55, *P* = 0.031). *n* = 6 per group for mBDNF; *n* = 6 and 5 in the adolescent and adult groups, respectively, for proBDNF.

(b) Both GluN1 and pGluN1 showed significant reductions from adolescence to adulthood (GluN1: *t(9)* = -3.48, *P* = 0.007; pGluN1: *t(8)* = -3.32, *P* = 0.011). *n* = 6 and 5 in the adolescent and adult groups, respectively, for GluN1; *n* = 5 per group for pGluN1.

(c) PSD-95 showed significant reduction from adolescent to adulthood (*t(10)* = -2.57, *P* = 0.028). *n* = 6 per group.

(d) No age differences were observed for the expression levels of GluA1. *n* = 6 per group.

**P* < 0.05, ***P* < 0.01, compared with the adult group. Ado, adolescence; Adult, adulthood.


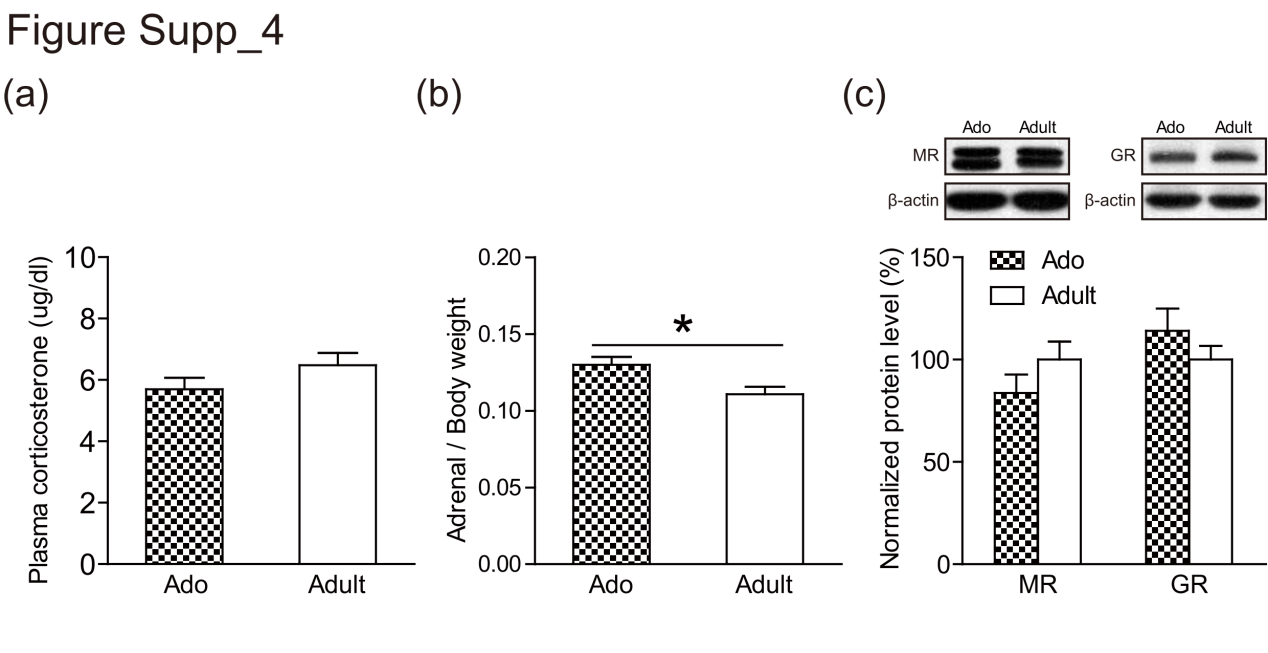


Figure S4. Age effects of neuroendocrine measures in control animals.

(a) Plasma corticosterone levels were comparable in adolescent and adult animals. *n* = 9 in the adolescent group and *n* = 8 in the adult group.

(b) Adolescent animals showed a significant increase in the relative weight of adrenal glands over body weight compared to adults (*t(18)* = 2.69, *P* = 0.015). *n* = 10 per group.

(c) No age differences were observed for the expression levels of MR and GR in rat hippocampus. *n* = 6 per group.

**P* < 0.05, compared with the adult group. Ado, adolescence; Adult, adulthood.
